# Novel Insights and Current Evidence for Mechanisms of Atherosclerosis: Mitochondrial Dynamics as a Potential Therapeutic Target

**DOI:** 10.3389/fcell.2021.673839

**Published:** 2021-07-07

**Authors:** Dan Li, Shengjie Yang, Yanwei Xing, Limin Pan, Ran Zhao, Yixi Zhao, Longtao Liu, Min Wu

**Affiliations:** ^1^Guang’an Men Hospital, China Academy of Chinese Medical Sciences, Beijing, China; ^2^Xiyuan Hospital, China Academy of Chinese Medical Sciences, Beijing, China

**Keywords:** mitochondria, morphology, fission, fusion, cytoskeleton, transport

## Abstract

Cardiovascular disease (CVD) is the main cause of death worldwide. Atherosclerosis is the underlying pathological basis of CVD. Mitochondrial homeostasis is maintained through the dynamic processes of fusion and fission. Mitochondria are involved in many cellular processes, such as steroid biosynthesis, calcium homeostasis, immune cell activation, redox signaling, apoptosis, and inflammation, among others. Under stress conditions, mitochondrial dynamics, mitochondrial cristae remodeling, and mitochondrial ROS (mitoROS) production increase, mitochondrial membrane potential (MMP) decreases, calcium homeostasis is imbalanced, and mitochondrial permeability transition pore open (mPTP) and release of mitochondrial DNA (mtDNA) are activated. mtDNA recognized by TLR9 can lead to NF-κB pathway activation and pro-inflammatory factor expression. At the same time, TLR9 can also activate NLRP3 inflammasomes and release interleukin, an event that eventually leads to tissue damage and inflammatory responses. In addition, mitochondrial dysfunction may amplify the activation of NLRP3 through the production of mitochondrial ROS, which together aggravate accumulating mitochondrial damage. In addition, mtDNA defects or gene mutation can lead to mitochondrial oxidative stress. Finally, obesity, diabetes, hypertension and aging are risk factors for the progression of CVD, which are closely related to mitochondrial dynamics. Mitochondrial dynamics may represent a new target in the treatment of atherosclerosis. Antioxidants, mitochondrial inhibitors, and various new therapies to correct mitochondrial dysfunction represent a few directions for future research on therapeutic intervention and amelioration of atherosclerosis.

## Introduction

Atherosclerosis is a chronic inflammatory condition caused by abnormal lipid metabolism, oxidative stress, endothelial injury and other factors and can involve large and medium-sized arteries throughout the body (Gisterå and Ketelhuth, 2018). Atherosclerotic cardiovascular disease (ASCVD) is a major cause of mortality in many industrialized societies (Commodore-Mensah et al., 2021). Lipid accumulation, local inflammatory responses, and endothelial injury are important factors in the development of atherosclerosis (Pham et al., 2021).

Over the past 20 years, studies have shown that mitochondrial dysfunction can lead to the occurrence and development of many diseases such as atherosclerosis (Sobenin et al., 2013a). Mitochondria are highly dynamic organelles that constantly produce adenosine triphosphate (ATP). Events, such as mitochondrial DNA (mtDNA) mutation, imbalance in calcium homeostasis, accumulation of oxidative stress products, and metabolic dysfunction are hallmarks of mitochondrial damage (Forte et al., 2019). When mitochondria are damaged or dysfunctional, energy production is limited and large quantities of reactive oxygen species (ROS) are produced. At the same time, mitochondria are vulnerable to damage from ROS. Cardiac cells, which are oxygen-hungry and mitochondria-rich, are also vulnerable to ROS damage. Studies have shown that ROS-mediated energy damage can induce systolic dysfunction of the heart (Luptak et al., 2019). In addition, ROS promote mutations and deletions in mtDNA (Li et al., 2021). Mitochondrial fusion can serve as a strategy to repair irreversibly damaged mitochondria, and at the same time, limit the accumulation of mtDNA mutations during aging. Irreversibly damaged mitochondria can also be repaired through fission (Yapa et al., 2021). Here we discuss the role of mitochondrial dynamics and its potential as a therapeutic target in this review.

## Mitochondrial Dynamics and Dysfunction in Atherosclerosis

**FIGURE 1:**
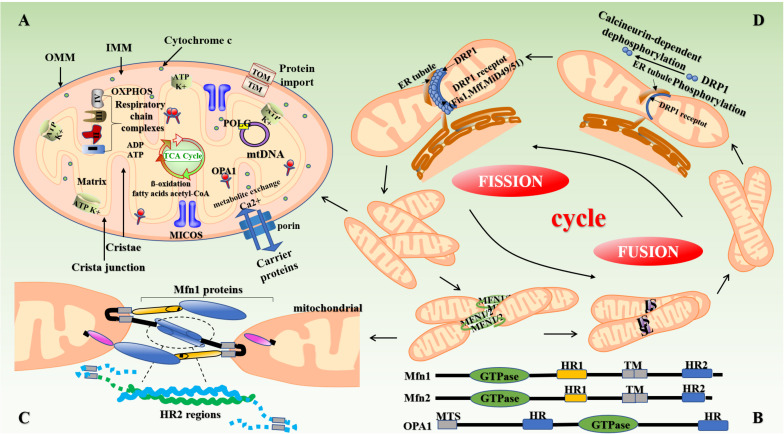
Structure of mitochondria and mitochondrial dynamics. **(A)** The mitochondrion is mainly composed of OMM, mitochondrial membrane gap, IMM, and the mitochondrial matrix. The intima folds inward to form mitochondrial cristae perpendicular to the mitochondrial long axis. Mitochondria produce reduced nicotinamide adenine dinucleotide (NADH) through the Krebs cycle, which is then oxidized and phosphorylated to release ATP. **(B,C)** Mitochondrial fusion involves three types of dyneins, namely MFN (Mfn1 and Mfn2), OPA1, and MSTO1. First, the transmembrane GTPases on the mitochondrial outer membrane, namely Mfn1 and Mfn2, fuse through the HR structure. Subsequently, OPA1-mediated IMM fusion occurs in the mitochondrial inner membrane. OPA1 also includes L-OPA1 and the S-OPA1 formed after the removal of L-OPA1 via action of proteolytic enzymes OMA1 and YME1L1. Mitochondrial mitosis is mediated mainly by Drp1. Drp1 is recruited into mitochondria by several ligand proteins (MFF, MIEF1/Mid51, and MIEF2/Mid49) that assemble into spiral fragments around the OMM, induce mitochondrial fission, and complete division by the transport of microtubules and actin. **(D)** The fusion and fission of mitochondria is a cyclic process. IMM, inner mitochondrial membrane; OMM, outer mitochondrial membrane; OPA1, optic atrophy protein-1; Drp1, dynamin-related protein; NADH, nicotinamide adenine dinucleotide.

Mitochondria are organelles with a double-membrane structure and are the main components involved in aerobic respiration in most eukaryotic cells (Navaratnarajah et al., 2021). The mitochondrial membrane comprises three layers. The outer layer is known as the outer mitochondrial membrane (OMM). The mitochondrial intima contains enzymes responsible for oxidative phosphorylation (OXPHOS), which are components of a multi-protein complex of five large electron-transport (respiratory) chains (Song et al., 2019; Figure 1A). Increased ROS levels result in mitochondrial dysfunction in vascular cells, aggravated endothelial injury and smooth muscle cell proliferation, and are responsible for inducing vascular atherosclerosis development and other pathological changes (Hughes et al., 2020). Furthermore, in the mitochondria, the activity of ion channels—which modulate Ca^2+^ signal transduction—is regulated by the free radicals generated through the respiratory chain functions, and these phenomenon subsequently affect biosynthesis and degradation reactions in various organisms (Gherardi et al., 2020). In addition, mitochondria are directly and closely related to other organelles such as the endoplasmic reticulum (Lackner, 2019). For example, mitochondria-associated endoplasmic reticulum membranes (MAMs) play an important role in atherosclerosis development, heart failure, and other diseases by participating in lipid and calcium (Ca^2+^) homeostasis, mitochondrial dynamics, inflammation, and apoptosis (Gao et al., 2020).

Studies have shown that the continuous fission and fusion of mitochondria are important for maintaining mitochondrial morphology and function (Kyriakoudi et al., 2021). Mammals contain two mitofusins, namely mitofusin (Mfn)-1 and Mfn2. These proteins contain two hydrophobic heptapeptide repeats, i.e., HR1 and HR2 at their N- and C-termini (located on both sides of the transmembrane domain) (Xin et al., 2021) (Figure 1B). In mammalian cells, mitochondrial fusion is mainly mediated by members of the of the GTPase protein family, i.e., Mfn1, Mfn2, and optic atropy-1 (OPA1) (Wolf et al., 2020). OPA1 and Mfn1 cooperate to enable organelle fusion. Transcript variants of *OPA1* encode two OPA1 protein isomers with different lengths, namely L-OPA1 and S-OPA1. As L-OPA1 has a better fusion efficiency than S-OPA1, its abnormal functioning can lead to reduced fusion activity, and thereby mitochondrial rupture and apoptosis (Wang et al., 2021a; Figure 1C). The collar structure comprising Drp1 polymer plays a central role in mitochondrial fission, and post-translational modification of Drp1 plays a major role in the formation of collar structures during mitochondrial fission (Breitzig et al., 2018; Figure 1D). Soluble substances can enter the mitochondria when the mPTP—located in the inner mitochondrial membrane—opens or closes, thereby affecting the MMP and inducing apoptosis (Du et al., 2021).

The expression of mitochondrial dynamin plays an important role in the development of atherosclerosis (Sharp and Archer, 2015). Chiong et al. (2014) found that the expression of Mfn2 is significantly reduced in the background of atherosclerosis in ApoE^–/–^ mice and is also involved in the pathogenesis of atherosclerosis. Heterozygous deletion of *OPA1* in mice also results in abnormal mitochondrial morphology, such as cleavage of the mitochondrial cristae (Hu et al., 2020). In some cases, inhibition of Drp1 expression can increase the depolarization of mitochondria in heart cells (Ikeda et al., 2015). Drp1-induced disturbances in mitochondrial homeostasis can cause a variety of complex vascular diseases through mechanisms, such as myocardial ischemia-reperfusion (I/R) injury, heart failure, and endothelial dysfunction (Morales et al., 2020).

## Novel Mechanistic Insights: From Mitochondrial Dynamics to Atherosclerosis

### Mitochondrial ROS-Induced Oxidative Stress in Atherosclerosis

The fusion and fission of mitochondria are closely related to mitochondrial function. ROS are a byproduct produced during mitochondrial respiration; when mitochondrial ROS (mitoROS) levels are disturbed, interactions involving the structure and function of mitochondria may eventuate (Forrester et al., 2018) and play important roles in the development of inflammatory and metabolic disorders (such as atherosclerosis and diabetes) (Hu et al., 2020). Drp1 can affect mitochondrial fission by regulating the levels of mitoROS and subsequent oxidative stress (Cid-Castro and Morán, 2021). In addition, ROS also regulate mitochondrial fusion. When ROMO1 (ROS regulatory protein 1) is inactivated, OPA1 expression is reduced, resulting in the remodeling of mitochondrial cristae and fragmented mitochondria (Norton et al., 2014). Studies have shown that high glucose levels can increase the activity of Drp1 in the mitochondria of endothelial cells, leading to mitochondrial fission and production of mitoROS. Mdivi-1 can reduce high glucose induced oxidative stress and injury to aortic cells (Wang et al., 2017b).

**FIGURE 2:**
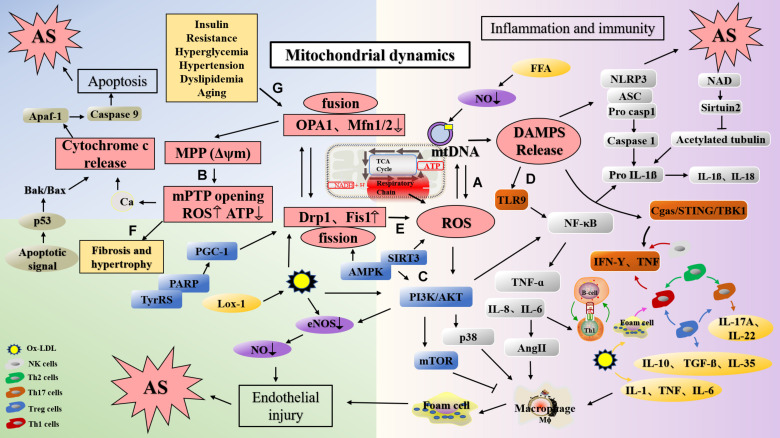
The mechanism of atherosclerosis formation caused via dysfunction of mitochondrial function and dynamics. **(A)** ROS produced in the respiratory chains of mitochondria can cause damage to mtDNA. **(B)** Ox-LDL induced the change of MMP, lead to Ca^2+^ influx, ROS production and mitochondrial damage. In addition, the decrease of MFN and OPA1 levels can also lead to the change of MMP and damage of mitochondria. **(C)** *PPAR* deletion decreases Mfn2 expression and PGC-1 expression, and leading to mitochondrial dysfunction; AMPK activates endothelial cells through the phosphatidyl inositol 3 kinase protein kinase B (PI3Kb) pathway, stimulates eNOS activation, and generates NO to protect endothelial cells; Mfn2 can inhibit the PI3K/Akt pathway by activating the mitochondrial apoptotic pathway, resulting in VSMC apoptosis. **(D)** DAMP promotes inflammation by activating PRRs. mtDNA can activate NF-κB and trigger TLR9 signaling pathway to mediate p38 pathway. Mitochondrial damage induces NLRP3 activation, and NLRP3 amplify the production of ROS. **(E)** Silencing Drp1 can inhibit mitochondrion fission, decrease ROS levels and inhibits smooth muscle cell migration. **(F)** Mfn2 deficiency leads to the increase of Ca^2+^ expression in cardiomyocytes, mitochondrial swelling, and eventually leads to cardiac hypertrophy. **(G)** Diabetes mellitus, insulin resistance, dyslipidemia, obesity, hypertension and aging may damage mitochondrial function, and lead to the development of atherosclerosis. mtDNA, mitochondrial DNA; Drp1, dynamin-related protein; Mfn1, mitofusin 1; Mfn2, mitofusin 2; OPA1, optic atropy-1; Fis1, mitochondrial fission protein 1; MMP (Δψm), mitochondrial membrane potential; mPTP, mitochondrial permeability transition pore open; Cyt c, cytochrome C; ROS, reactive oxygen species; LOX-1, lectin-type oxidized LDL receptor 1; ox-LDL, oxidized low density lipoprotein; DAMP, damage-associated molecular pattern; PI3K, phosphatidyl inositol 3; NF-κB, nuclear factor-κB; TyrRS-PARP1, tyrosyl transfer- RNA synthetase (TyrRS) and poly (ADP-ribose) polymerase 1 (PARP1).

Lipid accumulation is an important link in the formation of plaque during the early stages of atherosclerosis (Chistiakov et al., 2018), and increased ROS levels induce endothelial dysfunction, vascular inflammation, and accelerated accumulation of oxidized low density lipoprotein (ox-LDL) in the arterial wall, a phenomenon that promotes atherosclerosis (Naik and Dixit, 2011; Yu et al., 2017). As an activator of NLRP3, ox-LDL can induce alterations in MMP, which leads to the generation of mitoROS and activation of Ca^2+^ signals, calcium influx, and mitochondrial damage (Triantafilou et al., 2013). *In vitro* experiments have shown that lectin-type oxidized LDL receptor 1 (LOX-1), the main receptor for ox-LDL, expressed in response to lipopolysaccharide (LPS) induction, can lead to ROS production, mtDNA damage (Figure 2B), and the production of NLRP3 inflammasomes and play an important role in inflammatory diseases such as atherosclerosis (Ding et al., 2014). Studies have reported that ox-LDL and ROS can damage mitochondria, release mitoROS, induce the activation of NLRP3, elevate levels of IL-1β and IL-18, and cause inflammation (Huang et al., 2020; Markin et al., 2021). At the same time, ROS leads to endothelial nitric oxide synthase (eNOS) degradation by increasing the activity of mitochondrial arginase II (Suárez-Rivero et al., 2021). *In vivo* studies have found that Mfn2 inhibits ox-LDL-induced rabbit smooth muscle cell proliferation and reduces atherosclerotic plaques by regulating Akt and ERK phosphorylation (Guo et al., 2007b).

In addition to mitoROS produced by activity of the mitochondrial electron transport chain (ETC), NADPH oxidase (NOX), xanthine oxidase and cyclooxygenase can also release large amounts of ROS (Yeh et al., 2018). Various sources of ROS play an important role in angiogenesis (Fukai and Ushio-Fukai, 2020). Angiogenesis has a significant impact on the treatment of ischemic cardiovascular disease (CVD). Restoring intravascular perfusion by enhancing or inhibiting angiogenesis is an important means of treating peripheral arterial disease (PAD) caused by atherosclerosis (Simons et al., 2016). Mitochondria play a key role in angiogenic responses induced by growth factors such as VEGF (Guo et al., 2017) by regulating mitoROS-related activities (Wang et al., 2011). *In vivo* studies have shown that in presence of high glucose, the consumption of PDIA1 in endothelial cells can induce Drp1 sulfenylation at Cys^644^, promote mitochondrial fission, and increase ROS levels. Therefore, PDIA1 or the Cys oxidation-defective mutant Drp1 can promote angiogenesis in diabetic mice (Kim et al., 2018). Inhibition of Drp1 can result in the dysfunction of mitochondrial respiratory function (Ota et al., 2020).

### mtDNA Damage in Atherosclerosis

The mitochondrial genome comprises naked, independently encoded, double-stranded DNA molecules that exist mainly as small loops (in yeast and mammals) or linear molecules (protozoa) (Suzuki et al., 2011). mtDNA is the only DNA molecule that exists in the human cytoplasm. It is 16,569-basepair long and includes the heavy chain of the outer ring (high molecular weight) and the light chain of the inner ring (low molecular weight). mtDNA encodes 37 genes, among them, 13 protein-coding regions have been identified to play a role in maintaining normal cellular OXPHOS (Tang et al., 2020). High levels of mitochondrial mutation represent an important factor that leads to dysfunction of oxidative phosphorylation and energy metabolism and endothelial injury (Ueda et al., 2015). As opposed to genomic DNA, mtDNA in the mitochondrial matrix or inner membrane does not contain any histones and is free of structural protection; this DNA is in a state of continuous synthesis throughout the cell cycle, with poor stability and is in close proximity to the site where the electron transport system—that continuously produces ROS—is located. Therefore, mtDNA is more easily and extensively damaged (Ahmed et al., 2015).

In humans, mtDNA damage has been confirmed in atherosclerotic diseases, and may be attributed to the damage of this DNA by ROS produced by the adjacent respiratory chains (Muñoz-Carvajal and Sanhueza, 2020; Figure 2A). As such, mitochondria are the site of activation of the NRLP3 inflammasome. When mitochondria are dysfunctional, changes in the production of ROS, mtDNA release, cardiolipin, and NAD/NADH can activate NLRP3. mtDNA damage may therefore result in mitochondrial dysfunction and increased IL-1β levels through the aforementioned mechanisms of promoting atherosclerosis (Freigang et al., 2013). Furthermore, mtDNA damage leads to mitochondrial dysfunction, resulting in the removal of abnormal mitochondria, which may be detrimental to cell function under conditions of oxidative stress. Altered membrane potential in cells lacking MFN or OPA1 may cause mitochondrial damage, which can be compensated by the dynamic cycle of mitochondrial fusion and fission (Chen et al., 2007; Weaver et al., 2014). Studies have reported that mitochondrial fusion possesses a dual function, and that it not only protects the integrity of mtDNA, but also maintains the mtDNA function under conditions of mutational pressure. Therefore, mitochondrial fusion may have some compensatory effects on mtDNA mutation (Chen et al., 2010). Liu et al. (2020) found that overexpression of Mfn2 can increase MMP, enhance mitochondrial fusion, reduce mitoROS accumulation, activate the AMPK/SIRT3 signaling pathway, and prevent cardio-cerebrovascular ischemia/reperfusion (I/R) damage.

Furthermore, somatic mutations in the human mitochondrial genome may play a role in the development of atherosclerosis (Sazonova et al., 2017; Volobueva et al., 2019). Investigations on 12 aorta samples from male have revealed that compared with non-atherosclerotic intima, lipofibrous plaques have a high frequency of *MT-RNR1* A1555G, *MT-TL1* C3256T, *MT-CYB* G12315A, and *MT-TL2* G15059A (Sobenin et al., 2013b). In addition, compared with healthy vascular tissue, atherosclerotic plaques exhibit significant differences in the frequency of C3256T, T3336C, G12315A, and G14459A mutations (Sazonova et al., 2009). The C5178A mutation is more common in normal vascular tissues than in atherosclerotic plaques (Matsunaga et al., 2001).

### Pathways Related to Mitochondria in Atherosclerosis

Peroxisomal proliferator-activated receptor (PPAR) is a transcription factor activated by nuclear receptor superfamily ligands; it activates target genes and affects lipid metabolism, glucose homeostasis, cell proliferation, differentiation, apoptosis, and inflammatory responses (Teixeira et al., 2021). Previous studies have confirmed that PPARs are expressed in atherosclerotic plaques, suggesting that PPARs are closely related to atherosclerosis-related mechanisms such as transcriptional regulation of pro-inflammatory genes, e.g., cytokines, chemokines, vascular endothelial cell adhesion factors, and metallostromal proteases (Corona et al., 2014). Studies have shown that cardiac defects involving PPARs can also lead to abnormal mitochondrial morphology, excessive lipid deposition, and other phenotypic changes (Cheng et al., 2004). PPARα induces the downregulation of Mfn2 expression in high-fat or high-glucose treated cardiomyocytes by promoting mitochondrial fusion. Exogenous supply of Mfn2 in this background can restore MMP, inhibit mitochondrial oxidative stress, and improve mitochondrial function (Hu et al., 2019). Additionally, studies have revealed that *PPAR* deletion significantly decreases PGC-1 expression in C57BL/6J mice, thereby leading to mitochondrial dysfunction at the structural and functional levels (Zhou et al., 2016b). Yang et al. found that resveratrol could inhibit palmitic acid-induced damage to human umbilical vein endothelial cells (HUVECs), increase the expression of Mfn1, Mfn2, and OPA1, inhibit mitochondrial fragmentation, and reduce oxidative damage in endothelial cells by regulating mitochondrial fusion through the TyPRS-PARP1 signaling pathway (Yang et al., 2019; Figure 2C).

AMPK is a cellular energy receptor activated by AMP that affects sugar, fatty acid, and protein metabolism (Dehnavi et al., 2021). Importantly, AMPK activation can lead to inhibition of cell proliferation when cardiomyocytes and vascular smooth muscle cells (VSMCs) are in a state of ischemia and hypoxia, thus playing an important role in the regulation of cardiovascular diseases and in the prevention and treatment of atherosclerosis (Yan et al., 2018). Moreover, AMPK activates endothelial cells through the phosphatidyl inositol 3 kinase protein kinase B (PI3Kb) pathway, stimulates eNOS activation, and generates NO to further protect endothelial cells; these phenomenons play important roles in the prevention of atherosclerosis (Tousoulis et al., 2012; Xing et al., 2015; Figure 2C). Studies have shown that the AMPK-SIRT3 pathway also affects mitochondrial function (Karnewar et al., 2016). In addition to affecting cell function and metabolism, AMPK can also affect mitochondrial homeostasis by promoting mitochondrial fission. Antimycin A (complex I inhibitor) and antiretroviral drug antimycin A (complex III inhibitor) were discovered based on the theory that AMPK could induce mitochondrial fission (Toyama et al., 2016). Previous studies have shown that MFF is a new substrate of AMPK and plays an important role in AMPK-mediated regulation of mitochondrial morphology (Ducommun et al., 2015). Phosphorylation of AMPK-induced MFF by SAMP155 at Ser172, for example, is a potential mechanism used to explain mitochondrial fission due to diminished mitochondrial respiration. Additionally, MFF in human osteosarcoma cells has been shown to induce mitochondrial fission (Toyama et al., 2016). Notably, AMPK regulates mitochondrial fission through an autophagy-dependent Drp1 degradation mechanism. Observation of the aorta of PRKAA2/AMPKa2-deficient mice revealed that the number of autophagosomes in the aorta of PRKAA2/AMPKa2-deficient mice is significantly reduced, suggesting abnormal mitochondrial mitosis (Wang et al., 2017a).

The phosphoinositide 3-kinase/protein kinase B (PI3K/Akt) signaling pathway is known to regulate cell growth, differentiation, and proliferation (Shao et al., 2021). Studies have shown that knockout of a PI3Kγ subunit, i.e., P110γ can reduce the size of atherosclerotic plaques in ApoE^–/–^ and LDLr^–/–^ mice. Moreover, the Class IA PI3K signaling pathway can significantly reduce the levels of serum free fatty acids (FFA), cholesterol, and triglycerides in mice and inhibit the production of intracellular ROS (Wang et al., 2021b). Akt also plays an important role in glucose metabolism, apoptosis, cell proliferation, and other aspects of cell growth (Linton et al., 2019). Furthermore, Mfn2 can inhibit the PI3K/Akt pathway by activating the mitochondrial apoptotic pathway, resulting in increased mitochondrial outer membrane permeability and ultimately VSMC apoptosis (Guo et al., 2007a; Figure 2C). Fang et al. found that decreased Mfn2 expression might be related to pulmonary arterial smooth muscle cell (PASMC) proliferation under hypoxic conditions (Fang et al., 2016).

### Role of Mitochondria in Inflammation and Immunity Related in Atherosclerosis

Inflammation and immunity are inseparable in atherosclerosis, as the two influence each other to accelerate the progression of atherosclerosis (Saigusa et al., 2020). Atherosclerosis is not only an inflammatory disease, but it is also an autoimmune disorder. Additionally, atheromatous plaques and phenotypic changes in vascular cells are the main manifestations of atherosclerosis, and most of these immune responses are can be attributed to Th-1 cells (Kuznetsova et al., 2019). Furthermore, ox-LDL, ROS, and advanced glycation end products (AGEs) further aggravate the occurrence of inflammatory reactions and vulnerable plaque rupture events.

Inflammation is related to innate defense and tissue damage. Pattern recognition receptors (PRRs) are located on the surface of cell membranes or inside cells. They recognize and bind to pathogen-associated molecular patterns (PAMPs) and damage-associated molecular patterns (DAMPs) to trigger the inflammatory cascade in innate immunity (Zhang et al., 2010). PRRs, LPS receptors, Toll-like receptors (TLRs), and Nod like receptors (NLRs) play important roles in the pathogenesis of atherosclerosis (Shimada et al., 2012). In contrast, mtDNA that functions as a DAMP plays an important role in the inflammatory response. DAMPs can accumulate when mtDNA is damaged or degraded, and these promote inflammation by binding to—and activating—PRRs (Mathew et al., 2012; Picca et al., 2017). Studies have shown that cytokines produced by mitochondrial DAMPs play a key role in the inflammatory signaling pathway in atherosclerosis (Goossens et al., 2010; Yu et al., 2013; Tumurkhuu et al., 2016). NLRs are scaffold proteins that play key roles in regulating innate immune responses by triggering the NF-κB and mitogen-activated protein kinase (MAPK) signaling pathways, and by controlling caspase activation (Zhang et al., 2014). Mitochondrial antiviral signaling protein (MAVS) is a key signaling protein activated by viral RNA sensors RIG-1 and MDA5, which can promote gene expression by activating the NF-κB pathways (Loo and Gale, 2011). In addition, MAVS associates with NLRP3 and promotes its oligomerization, which leads to the activation of caspase-1 (Mohanty et al., 2019). It has recently been demonstrated that the activation of NLRP3 caused by the synthetic TLR7 ligand imiquimod is the result of the production of mitoROS induced by complex I of the respiratory redox chain and the quinone oxidoreductase NQO2 (Groß et al., 2016).

In recent years, increasing numbers of studies have demonstrated that mtDNA regulates the development of inflammation in disease states by activating the immune system (West and Shadel, 2017). In mice, inflammatory arthritis was induced upon the intra-articular injection of mtDNA, which induced the secretion of TNF by spleen cells; this was the first report on the immunological potential of mtDNA (Kepp et al., 2011). mtDNA can induce the activation of the NF-κB pathway and the release of TNF-α and IL-6 after being sensed by TLR9 (Zhong et al., 2016). mtDNA accumulation also results in the activation of caspase-1 and promotes the secretion of IL-1β and IL-18 in macrophages, thereby participating in a series of inflammatory responses (Mottis et al., 2019). Further, mtDNA activates the p38 and p42-44 MAPK pathways and chemotaxis of neutrophils to endothelial injury sites by triggering TLR9 signaling (Zhang et al., 2010). This induces the development of a range of inflammatory diseases, including rheumatoid arthritis, atherosclerosis, and non-alcoholic steatohepatitis. NLRP3 inflammasome, whose formation is triggered by mitochondrial damage and IRF3-signaling-induced endothelial inflammation also contributes to the progression of atherosclerosis (Mao et al., 2017). In addition, mitochondrial dysfunction may also amplify the activation of NLRP3 through the production of mitoROS (Figure 2D).

### Mitochondrial Associated Endothelial Injury and Smooth Muscle Proliferation in Atherosclerosis

Endothelial dysfunction leads to the development of atherosclerosis in patients with diabetes, and induces many changes in terms of mitochondrial dynamics and mitochondrial fission, and increases ROS production (Ago et al., 2010). Mitochondrial NOX4 promotes the production of ROS by mitochondria, which in turn can induce mitochondrial damage (Vendrov et al., 2015) and endothelial injury (Kim et al., 2016). Metformin can inhibit the expression of NOX4, reduce the production of ROS, and improve endothelial function (Cheng and Lanza-Jacoby, 2015; Victor et al., 2015). D-chiro inositol can inhibit the expression of Drp1, reduce the levels of NOX4, and enhance the production of NO in mouse aortic endothelial cells to protect against endothelial injury (Zhang et al., 2017). After hypoxia/reoxygenation (H/R) injury, ROS levels increase significantly, and ROS promote mitochondrial fission in myocardial endothelial cells through JNK-mediated phosphorylation of Drp1 (Chen et al., 2021b).

Epigenetic modifications induced in response to mtDNA damage have become a research hotspot in the domains of aging and atherosclerotic diseases (Schiano et al., 2015). *In vivo* studies have shown that even if ROS levels do not increase significantly, mtDNA damage can reduce mitochondrial respiration and ATP content in smooth muscle cells, promote apoptosis of VSMCs and aggravate atherosclerosis (Yu et al., 2014). After endothelial injury, proliferation, migration, and vascular remodeling of VSMCs are important for the rupture of atherosclerotic plaques, wherein the fission of mitochondria and deregulated secondary morphological functions play an important role (Wang et al., 2015; Hong et al., 2017). Once mitochondrion fission is inhibited by silencing Drp1, the protons leak across the mitochondrial inner membrane, resulting in decreased ROS levels in primary mouse smooth muscle cells, a phenomenon that inhibits smooth muscle cell migration (Wang et al., 2015; Figure 2E).

### Mitochondria-Related Fibrosis and Hypertrophy in Atherosclerosis

Mitochondrial damage is involved in myocardial cell loss and myocardial fibrosis, both of which eventually manifest as cardiac ischemia (Bonnans et al., 2014; Humeres and Frangogiannis, 2019; Nielsen et al., 2019). Atherosclerosis is the pathological basis for a variety of cardiovascular and cerebrovascular diseases, eventually leading to cardiac dysfunction (Jonsson and Bäckhed, 2017). Compared with cardiomyocytes, cardiac fibroblasts have lower mitochondrial respiratory function and expression of mitochondrial complexes I, II, III, IV, and V, and this is the main cause of cardiac fibrosis (Zhao et al., 2019). *In vivo* studies have found that mitochondrial respiratory chain complex dysfunction, mtDNA damage, increased ROS abundance, and secondary oxidative stress in myocardial infarction models lead to the activation of many protein kinases and transcription factors involved in hypertrophy signals (Rababa’h et al., 2018; Bugger and Pfeil, 2020).

STAT3 plays an important role in maintaining the physiological balance in the heart and protecting the heart from harm (Heusch et al., 2011; Gent et al., 2017; Kleinbongard et al., 2018). If myocardial cells are stimulated by H_2_O_2_ or treated with rotenone, mitochondrial function is impaired, and STAT3 signaling is inhibited. Cardiac fibroblasts also express STAT3. In cardiac fibroblasts, STAT3 activation promotes cardiac fibroblast proliferation (Haghikia et al., 2014) and hyaluronic acid accumulation during wound healing after acute myocardial infarction (Müller et al., 2014).

Mitochondrial dynamics play an important role in the development of cardiac hypertrophy (Jong et al., 2019). The MMP of cardiomyocytes in mice lacking Mfn2 is decreased and cells exhibit a certain degree of cardiac hypertrophy. The reason may be that the level of Ca^2+^ in mitochondria deficient in Mfn2 increases and the mitochondria swell. In addition, reduced cell death in cardiomyocytes lacking Mfn2 is related to the inhibition of mPTP (Qiu et al., 2020; Figure 2F). It has been reported that Drp1 expression is related to the pathogenesis of cardiac hypertrophy (Pennanen et al., 2014). In cardiomyocytes of hypertensive rats, high levels of ROS—associated with overexpression of Drp1—can activate calcineurin and CaMKII, and lead to aggravation of cardiac hypertrophy. Mdivi-1 can reduce the production of ROS and inhibit the expression of Drp1 (Hasan et al., 2018). L-2286 induced the translocation of mitochondrial Drp1, reduced Drp1 expression, inhibited mitochondrial fission, and reduced the number of mitochondrial cristae. At the same time, it increased the expression of OPA1 and Mfn2 to prevent the development of spontaneous left ventricular hypertrophy in rats (Ordog et al., 2021).

### Mitochondrial Dynamics and Risk Factors for Atherosclerosis

#### Diabetes Mellitus, Insulin Resistance, and Mitochondrial Dynamics

Atherosclerosis is the most common macrovascular complication of diabetes. Imbalance between oxidative and antioxidant systems *in vivo* leads to increased levels of ROS, a phenomenon that results in linear DNA strand breaks, an important factor in progression of atherosclerosis and functional damage to endothelial cells (Fetterman et al., 2016). Hyperglycemia can trigger this mechanism through the ETC, leading to endothelial cell injury and dysfunction (Forrester et al., 2018). Patients with diabetes exhibit altered mitochondrial dynamics and endothelial cell morphology; for example, the mitochondria of the immortalized endothelial cell line Eahy926 will rupture in the presence of high glucose (Paltauf-Doburzynska et al., 2004). *In vivo* studies showed that mitochondrial debris and ROS production increased in endothelial cells isolated from coronary arteries of diabetic mice (Makino et al., 2010). Furthermore, the expression of Drp1 and Fis1 is increased and the production of ROS augmented in HUVECs under high glucose conditions; Silencing of Drp1 can prevent the damage caused by insulin, calcium ionophores, and eNOS phosphorylation (Shenouda et al., 2011; Figure 2G).

Studies have shown that activation of various pro-inflammatory factors and signaling pathways during the development of atherosclerosis is closely related to insulin resistance (Boudina et al., 2009; Watanabe et al., 2014). The increase in ROS levels after endothelial injury can lead to the activation of the AMPK pathway, increase the level of eNOS, trigger insulin resistance, and promote the development of atherosclerosis (Förstermann et al., 2017). Insulin resistance also alters lipid and protein metabolism. Increased ROS and pro-inflammatory cytokine levels impair insulin signaling, activate the NF-κB pathway, perpetuate the inflammatory and oxidative environment, prolong insulin resistance, and to some extent prolong atherosclerosis. Changes in mitochondrial activity caused by mitochondrial number and functional abnormalities induced by abnormal Mfn2 expression are some of the characteristic features associated with insulin resistance (Peyravi et al., 2020).

#### Dyslipidemia, Obesity, and Mitochondrial Dynamics

Inappropriate changes in lifestyle and dietary habits and alterations to metabolism are responsible for the globally increasing incidence of obesity, even in developed countries (Blüher, 2019). As an important mechanism associated with obesity development, deregulated lipid metabolism also results in the development of atherosclerosis and other diseases (Laslett et al., 2012). When various factors lead to substantial LDL deposition, enhanced HDL transport capacity and increased macrophage-mediated lipid phagocytosis occur, and finally, foam cell deposition occurs in damaged areas of blood vessels, thereby leading to the subcutaneous formation of atherosclerotic plaques (Everts et al., 2014; Cader et al., 2016). Excessive LDL deposition can induce vascular cell apoptosis through a mitochondria-dependent pathway after oxidative modification (Nazzal et al., 2006). Ox-LDL mediates the opening of mPTP through the activation of cysteine proteases, and then the mitochondria release cytochrome C and activated caspase-3, thereby releasing interleukins and other inflammatory factors (Vindis et al., 2005; Figure 2G).

Aortic mtDNA damage and protein nitrification are significantly increased in ApoE^–/–^ mice exposed to secondhand smoke, suggesting that mtDNA damage caused by high cholesterol is one of the important mechanisms for the development of atherosclerosis. Additionally, Mfn2 is expressed at relatively low levels in the muscle tissues of obese people compared to those of lean individuals (Knight-Lozano et al., 2002). In some patients with extrahepatic cholestasis, Mfn2 expression in the liver is decreased, suggesting that Mfn2 plays an important role in regulating lipid metabolism and mitochondrial function (Chen et al., 2013).

#### Hypertension and Mitochondrial Dynamics

Mitochondria play an important role in maintaining the stability of arterial blood pressure by regulating the superoxide content and energy metabolism (Vaka et al., 2018). Energy metabolism disorders involving myocardial mitochondria may be an important mechanism in hypertension (Marshall et al., 2018). For example, the arterial blood pressure of SOD2-deficient mice is significantly increased with age under conditions of a high-salt diet, and oxidative stress in SOD2-deficient mice might explain this increase; this leads to inflammatory cell infiltration and promotes sodium retention (Rodriguez-Iturbe et al., 2007). Additionally, studies have found that cholesterol and blood pressure are elevated in patients aged approximately 30 years, and that the degree of elevation is related to age; further, mitochondrial tRNA mutation and decline in mitochondrial function may be important factors leading to the onset of the disorder in these patients (Wilson et al., 2004; Bernal-Mizrachi et al., 2005). In pulmonary arterial hypertension, mdivi-1 inhibits the mitochondrial fragmentation of PASMCs isolated under hypoxic conditions and improves the function of these cells, while overexpression of Drp1 increases mitochondrial fragmentation (Zhuan et al., 2020). Dikalova et al. (2020) studied an animal hypertension model involving SIRT3^–/–^ mice and found that decreased expression of mitochondrial deacetylase SIRT3 resulted in SOD2 inactivation and mitochondrial oxidative stress injury. Subsequently, mtDNA release activates inflammasomes and other inflammatory cells to stimulate accumulation of inflammatory cells, thereby damaging vascular endothelial cells and promoting the development of hypertension and vascular aging (Dikalova et al., 2020). Studies have shown that excessive ROS levels induced in response to altered mitochondrial morphology and apoptosis via dynein-mediated cytochrome C release are important mechanisms leading to the development of hypertension-associated left ventricular hypertrophy (López Farré and Casado, 2001; van Empel and De Windt, 2004; Figure 2G).

Abnormal mitochondrial function and changes in mtDNA are important factors affecting vasoconstriction (Zhou et al., 2016a). Liu et al. (2016) found that mitochondrial dynamics are closely related to the functional state of blood vessels. They also found that changes in arterial vascular state caused by changes in mitochondrial dynamics of smooth muscle cells caused changes in arterial blood pressure (Liu et al., 2016). Additionally, studies have shown that the mitochondria of pulmonary VSMCs affect respiratory function and oxidative metabolism by regulating intracellular calcium homeostasis and also affect pulmonary vascular contraction, which is a key factor in the pathogenesis of pulmonary hypertension (Tuder et al., 2012).

#### Aging and Mitochondrial Dynamics

A considerable number of studies have shown that the mechanisms involved in age-related cardiovascular dysfunction, such as mitochondrial fusion and fission disorder, mtDNA mutation, excessive ROS production, mitochondrial respiratory chain function, and metabolic dysfunction are closely related to mitochondrial functional homeostasis (Ames, 2004; Koltover, 2017). During cardiac aging, mitochondrial structures are destroyed and mitochondrial size increases (Duicu et al., 2013). Mitochondria promote fusion or inhibit fission to promote cell aging (Picca et al., 2018). MFN-1/2 and OPA1 modulate mitochondrial morphology in adult cardiomyocytes (Faelber et al., 2019). Sebastián et al. (2016) found that Mfn2 expression decreased with skeletal muscle aging and triggered increased numbers of damaged mitochondria. D’Amico et al. (2019) showed that MFF expression of RNA-binding protein Pumilio2 (PUM2) decreases with age, which further leads to reduced mitotic division and dysfunction.

The decline of mitochondrial energy metabolism in the heart is associated with aging, and aging leads to mtDNA damage, and ROS (Elorza and Soffia, 2021). Studies have shown that with age, mitochondrial volume increases, and a considerable amount of ROS is produced during oxidative phosphorylation (Figure 2G). mtDNA mutations are found in disease states in patients with age-related diseases, including chronic coronary artery disease (Phillips et al., 2014). Strutynska et al. (2016) found that the concentration of nitric oxide (NO) and hydrogen sulfide in the mitochondria of aged rats is decreased, while the level of ROS is increased, resulting in increased sensitivity of mPTP to calcium. Foote et al. (2018) observed aorta and carotid arteries in mice and found that at 44 weeks if age, carotid artery wall elasticity decreased, aortic collagen content and elastin fragmentation increased, arterial mtDNA copy number decreased, mitochondrial respiration decreased, and blood vessel aging accelerated.

## Anti-Atherosclerotic Treatment Targeting Through Mitochondria

Mitochondria are considered to be one of the main targets for the design and development of new drugs in CVD and other diseases (including cancer and neurological diseases), and represent a promising strategy to treat atherosclerosis by modulating the mitochondria (Zielonka et al., 2017).

### Diet and Lifestyle

#### Diet

Studies have shown that controlling cardiovascular risk factors by adjusting diet, correcting obesity and properly controlling blood sugar levels, can prevent mitochondrial stress and reduce mitochondrial damage (Stanzione et al., 2021). The increase in FFAs contributes to the activation of oxidative stress, mitochondrial stress and pro-inflammatory signals (Kaludercic and Di Lisa, 2020). *Trans*-fatty acids (TFAs), which are found in many fast foods and meats, are unsaturated fats. TFAs increase TG, LDL, and decrease LDL particle size and HDL levels. TFAs also increase pro-inflammatory cytokine abundance, inducing endothelial dysfunction and insulin resistance (Micha and Mozaffarian, 2009). Artificial TFAs are associated with an increased risk of atherosclerosis and CV events (Valenzuela et al., 2019). The level of plasma FFA was increased with carotid atherosclerotic plaque in 320 patients with type 2 diabetes mellitus (T2DM) through carotid artery ultrasound examination and reporting, so reducing plasma FFA levels may be an effective way to reduce T2DM (Tibaut et al., 2019). Previous studies have reported that FFA can increase NO production, damage mtDNA and induce apoptosis (Li et al., 2015a).

#### Lifestyle

In addition, sedentary time is an independent risk factor for atherosclerosis and CVD, and at least one-third of deaths from coronary heart disease or T2DM are associated with sedentary time (Thijssen et al., 2010; Fletcher et al., 2018). In turn, exercise enhances endothelial function, protects against oxidative stress and inflammation, reduces the levels of TG, ApoB, and LDL, and increases HDL (Cai et al., 2018). Studies have shown that long-term aerobic exercise can reduce the formation of ROS and mitochondrial swelling in aortic endothelial cells of aged rats, increase the content of mtDNA, and reduce the vascular sclerosis and endothelial dysfunction caused by aging (Gu et al., 2014). However, it should be noted that excessive and overloaded exercise can also induce mitochondrial disorders, cause heart abnormalities, chronic fatigue syndrome and other diseases (Ostojic, 2016). Studies have confirmed that strenuous exercise can cause muscle dysfunction and increase mitochondrion fission (Pataky and Nair, 2021).

### Anti-atherosclerotic Drugs Targeting Mitochondria

#### Antioxidants

Selective mitochondrial-targeting drugs such as mitochondrial antioxidants are being tested in preclinical and clinical trials (Kiyuna et al., 2018). Some natural Chinese medicine ingredients with antioxidant effects have also been gradually discovered (Table 1). For example, luteolin exhibits antioxidant properties in HUVECs that significantly reverse the symptoms of oxidative stress in atherosclerosis (Wu et al., 2018). Resveratrol has been shown to promote mitochondrial fusion and can improve endothelial cells by maintaining mitochondrial membrane proteins and reducing ROS, and may be used in the prevention of atherosclerosis (Yu et al., 2019). Studies have found that Ilexgenin A inhibits palmitate-induced Drp1 expression and mitochondrial fission by regulating proteases, reduces the production of ROS and inflammatory factors, improves endothelial dysfunction, and reduces atherosclerosis (Zhu et al., 2019). Salidroside is considered to be an antioxidant with anti-cardiovascular and vascular protective effects. It can inhibit VSMC proliferation, Drp1 expression and oxidative stress, and up-regulate Mfn2 expression, which may improve the proliferation of VSMCs induced by high glucose (Zhuang et al., 2017). Corylin, a flavonoid compound, inhibits the proliferation of VSMCs induced by platelet-derived growth factor-BB (PDGF-BB) by regulating mTOR/Drp1, and reduces atherosclerotic lesions in ApoE^–/–^ mice (Chen et al., 2020).

**TABLE 1:** Natural compounds target mitochondrial to ameliorate atherosclerosis.

Natural compounds	Sources	Cell types	Changes to mitochondrial	Effects on mitochondria and atherosclerosis	References
Resveratrol	*Polygonum cuspidatum*	HUVECs	Mfn1, Mfn2 and OPA1↑, fission↓, ROS↓	Attenuated endothelial oxidative injury by regulating mitochondrial fusion, inhibiting mitochondrial fission via TyrRS-PARP1 signaling pathway.	Yang et al., 2019
Salidroside	Component of *Rhodiola rosea*	VSMCs isolated from aorta of male Sprague Dawley (SD) rats	Drp1↓,Mfn2↑, fission↓, ROS and NADPH↓	Inhibits high glucose induced proliferation of VSMCs by inhibiting mitochondrial fission and regulating oxidative stress	Zhuang et al., 2017
Corylin	*Psoralea corylifolia* L. (Fabaceae)	HUVECs A7r5 VSMC and RAW264.7 cells	Drp1 and Drp1 phosphorylation↓, fission↓, ROS↓	Inhibited the proliferation and migration of mammalian VSMC, in which rapamycin target protein (mTOR)/Dynamin-1 like protein 1 (Drp1) played an important role.	Chen et al., 2020
Ilexgenin A	*Ilex hainanensis Merr.*	RAECs and HUVECs	Drp1↓, fission↓ ROS↓, NO↑	Promote the expression of PSMB5, inhibit ROS production and Drp1 in a Nrf2 dependent manner, thereby inhibiting mitochondrial fission and improving endothelial dysfunction.	Zhu et al., 2019
Berberine	*Coptis chinensis* Franch	mouse podocytes	Drp1↓, MFF↓, Fis1, fission↓, ROS↓	Improve the mitochondrial damage of glomerular podocytes in DKD mice by inhibiting Drp1, Fis1 and mitochondrial fission.	Qin et al., 2019
Quercetin	Component of hawthorn	Calcifying VSMCs	Drp1↓, fission↓, ROS↓	Improve mitochondrial cristae rupture, inhibit mitochondrial fission, reduce ROS production, reduce apoptosis of VSMCs, thus alleviate adenine induced aortic calcification in rats.	Cui et al., 2017
Vitexin	Component of hawthorn	H9c2 cells	Mfn2↑, Drp1↓, fission↓, ROS↓, inhibited the release of Cyt-c, MMP(Δψm)↑, ATP↑	Protects H9c2 cells from I/R-induced mitochondrial dysfunction and significantly reduces ROS level by alleviating myocardial I/R injury in rats.	Xue et al., 2020
Crocin	Ingredient of saffron	Cells from muscle tissue of rats	Mfn2↑, Drp1↓	Change insulin resistance index and glucose homeostasis in diabetes by improving mitochondrial fusion and fission indices.	Peyravi et al., 2020
Baicalin	*Baikal Skullcap*	Rat pheochromocytoma PC12 cells	Drp1↓, fission↓, Mfn2↑, Drp-1 Ser637 phosphorylation↑, MMP(Δψm)↑, ROS↓,	Protected against hyperglycemia aggravated I/R injury by regulating mitochondrial functions in a manner dependent on AMPK.	Li et al., 2017

*HUVECs, human umbilical vein endothelial cells; RAECs, rat aortic endothelial cells; VSMCs, vascular smooth muscle cells; Drp1, dynamin-related protein; Mfn1, mitofusin 1; Mfn2, mitofusin 2; OPA1, optic atropy-1; ROS, reactive oxygen species; NADPH, nicotinamide adenine dinucleotide phosphate; MFF, mitochondrial fission protein; Fis1, mitochondrial fission protein 1; Cyt c, cytochrome C; MMP (Δψm), mitochondrial membrane potential; DKD, diabetic kidney disease; I/R, ischemia/reperfusion.*

#### Mitochondrial Homeostasis Regulator

Therapeutic strategies for maintaining mitochondrial homeostasis are already under study. MitoTEMPO, a mitochondrial-targeted SOD mimic, can reduce mitochondrial superoxide anions in high-fat diet mice, reduce the production of mitoROS, and prevent cardiomyocyte hypertrophy in the hearts of diabetic mice (Ni et al., 2016). Currently, specific inhibitors of mitochondrial fusion (*M*-hydrazone) and fission (MDIVI-1 and P110) are under investigation (Cassidy-Stone et al., 2008; Qi et al., 2013). mtDNA is an important cause of ROS production and mitochondrial damage. Mitochondrial miRNA is involved in the post-transcriptional regulation and metabolism of mitochondrial gene expression, ROS production and lipid metabolism, and can lead to abnormal mitochondrial function and increased oxidative stress, such as miR-484 inhibition of Fis1 expression. In addition, related research regarding mtDNA and mitochondrial miRNA may be a future direction for diagnosis and treatment of mitochondrial-related diseases (Song et al., 2019).

#### AMPK Regulator

Some drugs are aimed at regulating the levels of mitochondrial fusion and fission proteins by activating AMPK kinase, inhibiting ROS and inflammation and thereby improving endothelium, and prevent and treat atherosclerosis (Apostolova et al., 2020). For example, Coenzyme Q10 (CoQ10) is one of the components of the mitochondrial respiratory chain, which performs electron transfer, reduces oxidative stress damage and improves mitochondrial function. *In vivo* studies have shown that CoQ10 may negatively regulate YAP by activating AMPK and promote the expression of OPA1 to improve mitochondrial function, inhibit ROS production, and improve atherosclerosis (Xie et al., 2020). Thiazolidinediones such as pioglitazone as PPARγ inhibitors can activate AMPK and increase the expression of genes related to mitochondrial function. Studies have shown that AMPK activation regulates Drp1 phosphorylation to help inhibit the activation of mitochondrial ROS and TXNIP/NLRP3, thereby improving endothelial dysfunction (Li et al., 2015b).

#### NLRP3 Regulator

In atherosclerosis, oxidative stress and mitochondrial dysfunction are important mechanisms leading to NLRP3 activation. NLRP3 activation is closely related to mitochondrial damage (Ding et al., 2014). Fatty acid-mediated mitochondrial cartilage uncoupling promotes the release of NLRP3-dependent interleukin-1α (IL-1α) and aggravates the progression of atherosclerosis (Freigang et al., 2013). Both *in vivo* and *in vitro* studies have shown that Drp1-mediated mitochondrial fission is the cause of the activation of NADPH and NLRP3 inflammasomes in endothelial cells (Li et al., 2016). Statins mainly act by inhibiting 3-hydroxymethyl-3-glutaryl CoA (HMG-COA) reductase to reduce intracellular cholesterol biosynthesis. Approximately 40% of patients who fail to achieve their target levels after high doses of statins are treated with a combination of statins and other drugs (Boekholdt et al., 2014). In addition to lowering cholesterol, statins can also improve the endothelium through antioxidant activity to play an anti-atherosclerotic effect (Oesterle et al., 2017). *In vivo* studies found that mitochondrial ROS levels in mice treated with rosuvastatin are decreased, NLRP3, caspase-1 and IL-β levels decreased, mitochondrial damage was reduced, and myocardial fibrosis and infarct size were significantly reduced (Chen et al., 2021a).

## Conclusion and Perspectives

Atherosclerosis is a disease caused by multiple complex factors. A high-fat and high-calorie diet leads to the deposition of lipid particles, and ox-LDL produces a series of complex oxidative stress and inflammatory responses to endothelial stimulation, eventually forming foam cells and typical atheromatous plaques. In recent years, an increasing number of studies have shown that atherosclerosis may be related to mitochondrial fusion and fission. Cardiomyocytes consume substantial amounts of energy, and mitochondria produce ATP through oxidative phosphorylation. The dynamic homeostasis of mitochondria is essential to ensure normal functioning. Multiple studies have shown that mitochondrial dynamic dysfunction, such as mitochondrial over-division due to the absence of the fusion protein Mfn2 or overexpression of Drp1, can lead to CVD progression. The mechanisms involved in atherosclerosis may be closely related to mitochondrial fusion and fission.

In addition to lifestyle improvements and drugs such as statins, new types of antioxidants and mitochondrial regulators such as mdivi-1 have become research hotspots for the treatment of atherosclerosis. Exploration of treatment options for atherosclerosis is warranted; however, this is difficult because only a few classes of drugs are available for treatment, lipid-lowering therapy standards have not been agreed upon, and the cost of new drugs remains unaffordable. Moreover, the mechanisms involved in mitochondrial dynamics are relatively complex and their study is limited as the models are affected by many factors. Therefore, studies on the role of mitochondrial dynamics in atherosclerosis are at the basic research stage and lacks validation based on large-scale clinical studies. While mitochondrial dynamic homeostasis may play a role in atherosclerotic therapy, this hypothesis needs further confirmation.

## Author Contributions

MW and LL designed and directed the manuscript. DL wrote the manuscript. LP and SY revised the manuscript. RZ searched the literature. YX and YZ aided in the design of the illustrations. All the authors approved the manuscript for publication.

## Conflict of Interest

The authors declare that the research was conducted in the absence of any commercial or financial relationships that could be construed as a potential conflict of interest.
